# Novel and Facile Colorimetric Detection of Reducing Sugars in Foods via In Situ Formed Gelatin-Capped Silver Nanoparticles

**DOI:** 10.3390/polym15051086

**Published:** 2023-02-21

**Authors:** Reda M. El-Shishtawy, Yasser M. Al Angari, Maha M. Alotaibi, Yaaser Q. Almulaiky

**Affiliations:** 1 Chemistry Department, Faculty of Science, King Abdulaziz University, Jeddah 21589, Saudi Arabia; 2 Department of Chemistry, College of Science and Arts at Khulis, University of Jeddah, Jeddah 21921, Saudi Arabia

**Keywords:** reducing sugar, spectrophotometry, gelatin-AgNPs, DNS, maltose, glucose

## Abstract

The evolution of green technology for the simple and ecological formation of silver nanoparticles (AgNPs) inspired the present work for simple and efficient detection of reducing sugars (RS) in foods. The proposed method relies on gelatin as the capping and stabilizing agent and the analyte (RS) as the reducing agent. This work may attract significant attention, especially in the industry, for testing the sugar content using gelatin-capped silver nanoparticles as it not only detects the sugar in food, but also determines the content (%), which could be an alternative technique to the conventionally used DNS colorimetric method. For this purpose, a certain amount of maltose was mixed with a gelatin-silver nitrate. Different conditions that may affect the color changes at 434 nm owing to the in situ formed AgNPs, such as gelatin-silver nitrate ratio, PH, time, and temperature, have been investigated. The 1:3 mg/mg ratio of gelatin-silver nitrate dissolved in 10 mL distilled water was most effective in color formation. The development of AgNPs color increases within 8–10 min at PH 8.5 as the selected optimum value and at the optimum temperature of 90 °C for the evolution of the gelatin-silver reagent’s redox reaction. The gelatin-silver reagent showed a fast response (less than 10 min) with a detection limit for maltose at 46.67 µM. In addition, the selectivity of maltose was checked in the presence of starch and after its hydrolysis with α-amylase. Compared with the conventionally used dinitrosalicylic acid (DNS) colorimetric method, the proposed method could be applied to commercial fresh apple juice, watermelon, and honey to prove its viability for detecting RS in fruits; the total reducing sugar content was 287, 165, and 751 mg/g, respectively.

## 1. Introduction

Carbohydrases (glycoside hydrolases or *O*-glycosidases) are a significant class of enzymes that hydrolyze polysaccharides and low-molecular-weight glycosides. They belong to the hydrolase family of enzymes. Amylase, cellulases, xylanases, mannanases, pectinases, chitinases, and other carbohydrases are categorized according to their selectivity toward natural glycoside substrates. In biotechnology, many carbohydrates have found extensive applications [1]. The majority of carbohydrase activity measurement techniques depend on the examination of reducing sugars (RS) generated as a result of the enzymatic cleavage of a glycosidic bond between two carbohydrates or between a carbohydrate and a noncarbohydrate component [2,3]. In addition to performing particular roles in vital processes, carbohydrates are significant macronutrients that function as a primary source of energy in human nutrition [4]. Deoxyribose and ribose in nucleic acids structure, lactose in milk, and galactose in some oils are a few examples. The four categories of carbohydrates are monosaccharides, disaccharides, oligosaccharides, and polysaccharides. Free-form monosaccharides and disaccharides are commonly referred to as sugars, and depending on how they react chemically, they can be divided into reducing and non-reducing sugars [5]. Sugars are a marker for several nutritional qualities, including flavor, naturalness, and taste [6]. Reducing sugar is essential for biological samples, such as tissue, blood, plasma, and serum. European law regulates the sugar level of some products and beverages [7]. Since sugars are known to be crucial in developing severe diseases (such as obesity and diabetes), determining their identity is a complex analytical challenge. Sugar measurement is required in several intricate biological systems and food and beverage matrices. Soft drinks with added sugar should be given extra attention because they are the primary source of calories in the American diet and raise the risk of obesity [8]. Advanced analytical methods for determining carbohydrate concentrations have been developed as a result of the wide variety of carbohydrates present in these areas, including capillary electrophoresis [9], chromatography [10], infrared (IR) spectroscopy [11], nuclear magnetic resonance (NMR) spectroscopy [12], and light scattering detection [13]. These techniques demand a substantial financial investment, sophisticated analytical abilities, and effort. The colorimetric method, based on a redox reaction in which a reducing sugar acts as the reductant and the reagent as the oxidant leading to the production of color that could be measured by the spectrophotometric method, is one of the most flexible, reasonably simple, and affordable methods for determining reducing sugar. Different techniques for evaluating RS have been used in carbohydrate activity estimations. The Somogyi–Nelson [2], phenol-sulfuric acid [14], anthrone-sulfuric acid [15], Fehling [16], and dinitrosalicylic acid (DNS) [17] methods were evaluated for the determination of RS. The Fehling approach involves several analysis steps (including precipitation and titration). The phenol–sulfuric acid method has a variety of significant limitations. Multiple health risks are associated with phenol employed in this approach. The prolonged or repeated inhalation of phenol fumes brings on lung edema. Long-term phenol exposure seriously impacts the central nervous system [18]. The Nelson–Somogyi assay and the 3,5-dinitrosalicylic acid (DNS) assay described are the most popular methods used by many researchers. Although the DNS assay is known to be approximately 10 times less sensitive than the Nelson–Somogyi assay and it does not provide stoichiometric data with oligosaccharides, giving significantly higher values of RS than the actual number of hemiacetals reducing groups [19,20,21], it has been recommended by the International Union of Pure and Applied Chemistry (IUPAC) commission on biotechnology for measuring standard cellulase activities against filter paper and carboxymethylcellulose (CMC) [22]. Our goal was to develop a visible spectrophotometric method for quantifying RS with high sensitivity. We have developed a new and simple method for determining reducing sugar. Gelatin-silver reagent was found to be suitable for the determination of reducing sugar. In previous literature, gelatin-silver nanoparticles were utilized for several purposes. Gelatin-silver nanoparticles were used as antimicrobial composite films [23], and gelatin-silver nanoparticles coating for polycaprolactone was used for wound healing [24]. Composite films made of chitosan/gelatin-silver nanoparticles were used in biodegradable food packaging [25]. Gelatin, an abundant biopolymer, is both biocompatible and biodegradable. It can be extracted from animal tissues such as muscle, bone, and skin. Gelatin is widely employed in pharmaceutical, cosmetics, food, and medical applications due to its natural abundance and inherent biodegradability in physiological conditions [26]. The most common nanoparticles used in food and other industries are silver nanoparticles. A nanoparticle is described as a tiny item or particle that behaves as a whole unit in terms of its transport and properties. Nanotechnology makes use of the fact that when the size of a solid material shrinks, its surface area grows, enhancing its reactivity and quantum-related phenomena. Nanomaterials’ physical and chemical characteristics can change dramatically from those of the same substance in its bulk size [27]. Consumer products, food technology, textiles/fabrics, and the medical industries are all interested in silver NP due to its chemical and biological qualities. Silver NP also has special optical and physical characteristics that are not found in bulk silver and are said to offer a lot of potential in medicinal applications [27]. Generally, the formation of nanoparticles such as AgNPs has been made possible ecologically using reducing sugars such as glucose [28,29,30,31,32]. As a capping agent, gelatin in the presence of glucose was used for the green preparation of AgNPs [33]. The appearance of visible color due to the surface plasmon resonance (SPR) of nanoparticles has caught the attention of many researchers. The AgNPs SPR appear around 400 nm and, thus, could be applied as an analytical tool for detecting grallic acid [34], o-phenylenediamine [35], formaldehyde [36], and RS using Tollens’ reagent [37]. Gold nanoparticles were also used for the determination of RS [38]. 

Given the benefits that AgNPs provide, such as their high SPR across a broad spectrum [39], low cost, and environmentally friendly production, it was intended in this work to exploit the SPR of AgNPs that can be formed via a redox process with RS for its detection in the presence of gelatin as a capping agent for the in situ formed AgNPs.

## 2. Experimental

### 2.1. Materials

Silver nitrate, gelatin, maltose, starch, tris(hydroxymethyl)aminomethane, nitric acid, α-amylase from porcine pancreas, and 3,5-dinitrosalicylic acid (DNS) were purchased from Sigma-Aldrich (Saint Louis, MO, USA) and then used as obtained. Watermelon, apple, and honey juice samples were purchased from local market, Jeddah, KSA.

### 2.2. Effect of Gelatin-Silver Reagent Ratio

To determine the gelatin-silver nitrate ratio for effective color formation, the gelatin-silver nitrate reagent was prepared in 10 mL distilled water at a ratio weight by weight (for example, 100 mg/100 mg for 1:1 ratio) 1:1, 2:1, 1:3, 1:2, and 2:2. The conditions at which the best chemical treatment yield obtained was 1:3 gelatin-silver nitrate. A 1 mL of reaction mixture contained 250 µL maltose (0.1 mM), 250 µL Tris–HNO_3_ buffer PH 8.5 (0.2 M) (prepared by 0.2 M of tris(hydroxymethyl)aminomethane then adjust PH by dilute HNO_3_ to the appropriate PH), and 500 µL gelatin-silver reagent. The reaction mixture was incubated for 10 min at 90 °C in a boiling water bath before cooling then the absorbance was recorded at 434 nm. The recorded optical density (OD) at 434 nm was used to determine the relative OD (%).

Relative OD (%) = (OD_x_/OD_max_) × 100 (1)

where OD_max_ is the maximum OD and OD_x_ is OD for a sample with OD less than the maximum OD.

### 2.3. Effect of Time

To determine the effect of time on the evolution of color, a 1 mL of reaction mixture contained 250 µL maltose (0.1 mM), 250 µL of 0.2 M Tris–HNO_3_ buffer PH 8.5, and 500 µL gelatin-silver reagent (1:3). The reaction mixture was incubated for different times (2–20 min) at 90 °C in a boiling water bath then cooled to room temperature to measure the absorbance at 434 nm. The recorded OD was used to determine the relative OD (%).

### 2.4. Effect of PH on the Silver-Gelatin Reagent

The following buffers were used to determine the optimal PH, 50 mM Tris–HNO_3_ (PH 6.0–8.5). A 1 mL of reaction mixture contained 250 µL maltose (0.1 mM), 250 µL of different Tris–HNO_3_ buffers (0.2 M), and 500 µL gelatin-silver reagent (1:3). The reaction mixture was incubated for 10 min at 90 °C in a boiling water bath before cooling then the absorbance was recorded at 434 nm. The recorded OD at 434 nm was used to determine the relative OD (%). 

### 2.5. Effect of Temperature

Different scales (30–90 °C) were applied to the reaction mixture containing 250 µL maltose (0.1 mM), temperature 250 µL of different Tris–HNO_3_ buffers, and 500 µL gelatin-silver reagent (1:3) to assess the impact of temperature on the gelatin-silver reagent. The reaction mixture was incubated for 10 min at different temperature scales in a boiling water bath before cooling then the absorbance was recorded at 434 nm. The recorded OD at 434 nm was used to determine the relative OD (%).

### 2.6. Maltose Selectivity

The gelatin-silver method was validated as selective toward reducing sugar as follows; in the Eppendorf tube, 185 µL of maltose (0.1 mM) was added to 185 µL of starch (1%) and 125 µL of Tris-HNO_3_ buffer (0.2 M, PH 8.5). In another tube, 185 µL of maltose (0.1 mM) was added to 190 µL distilled water and 125 µL Tris-HNO_3_ buffer. The third tube contained 185 µL of starch (1%), 190 µL distilled water, and 125 µL Tris-HNO_3_ buffer. A volume of 500 µL of gelatin-silver reagent (1:3) was added to all tubes. It was then incubated for 10 min at 90 °C in a boiling water bath and then cooled to room temperature to measure the absorbance at 434 nm.

### 2.7. Hydrolysis of Starch with α-Amylase

The hydrolysis of starch was carried out using α-amylase to ascertain the impact of gelatin-silver reagent on the reducing sugar content generated by carbohydrase enzymes. Thus, in the Eppendorf tube, 10 units of α-amylase were incubated for 30 min with 0.06, 1.25, and 1.9% starch (1 mL prepared in 50 mM Tris–HNO_3_ buffers, PH 7.0) at 37 °C. After that, 1 mL of gelatin-silver reagent (1:3) was added and heat for 10 min at 90 °C to help develop the color. After cooling, the absorbance was recorded at 434 nm. One unit of α-amylase activity was defined as the amount of enzyme producing 1 μmol reducing sugar as maltose per min under the standard assay conditions [17].

### 2.8. Effect of Maltose Concentration and Detection Limit

Different concentrations of maltose (0.2–1.2 mM) were applied, and OD values were recorded following the reaction condition described above. A sample of different concentrations of maltose, 0.2 M Tris–HNO_3_ buffer PH 8.5, and 500 µL gelatin-silver reagent (1:3) was incubated for 10 min at 90 °C in a boiling water bath before cooling. Then the absorbance was recorded at 434 nm ten times to determine the standard deviation from which and the linear relation of maltose concentration versus OD, the limit of detection (LOD), was obtained. 

### 2.9. Real Samples Analysis

Watermelon, apple, and honey juice samples were used as models to determine the effect of gelatin-silver reagent on reducing sugar content in some food samples. The watermelon, apple, and honey samples were supplied from a local market in Jeddah. The fruit of the watermelon and apple were rinsed, skinned, and eliminated waste. One gram of watermelon and apple flesh was crushed separately on a glass grater. The watermelon and apple juices were extracted from the pulps by centrifugation at 6000 rpm for 5 min and filtering using a PTFE filter with a pore size of 0.45 μm. The honey sample was generated by weighing 1.0 g of honey, homogenizing it with distilled water, and then diluting it to 100 mL with distilled water. Reducing sugar was determined as follows: 250 µL of each sample was mixed with 250 µL of different Tris–HNO_3_ buffers (0.2 M, PH 8.5) and 500 µL gelatin-silver reagent (1:3). The reaction mixture was incubated for 10 min at 90 °C in a boiling water bath before cooling then the absorbance was recorded at 434 nm. A standard curve of maltose concentrations was used to determine the total reducing sugar contents. The total reducing sugar contents were recorded as mg maltose Eq. g^−1^ of the studied sample.

DNS reagent was utilized to determine each sample’s total reducing sugar contents and compare it with our method. DNS reagent was prepared according to the Miller method [17] as follows: 20 g of potassium sodium tartrate was dissolved in 20 mL distilled water and stirred to totally dissolved, then sodium hydroxide (1 g, 20 mL) was added, followed by 1 g of 3,5-dinitrosalicylic acid prepared in 60 mL of distilled water. While the solution was mixed by magnetic stirrer with a hot plate at 90–95 °C, 50 mg of sodium sulfide was added followed 200 mg of phenol. After the components were dissolved entirely, the final solution was filtered with filter paper, then transferred the solution in dark glass bottles and stored at ambient temperature. Reducing sugar was determined according to DNS reagent: 250 µL of each sample was mixed with 250 µL of different Tris–HCl buffers (0.2 M, PH 7.0) and 500 µL DNS reagent. The reaction mixture was incubated for 10 min at 95 °C in a boiling water bath before cooling then the absorbance was recorded at 560 nm. A standard maltose concentration curve was used to determine the total reducing sugar contents.

## 3. Results and Discussion

### 3.1. Gelatin-Silver Method Optimization

**Figure 1:**
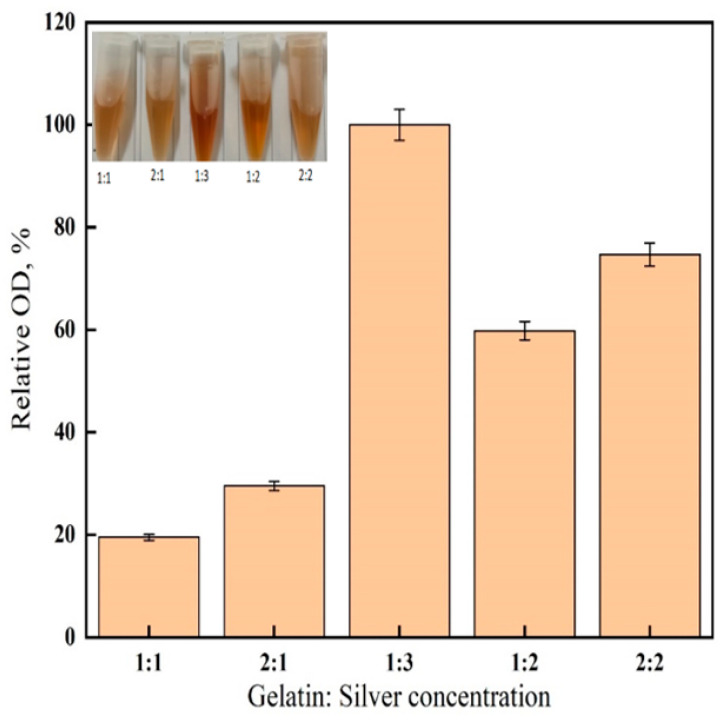
Effect of gelatin- silver reagent ratio on the color evolution. Conditions: A 1 mL of the reaction mixture contained 250 µL maltose (0.1 mM), 250 µL Tris–HNO_3_ buffer PH 8.5 (0.2 M), 500 µL gelatin-silver reagent (1:1, 2:1, 1:3, 1:2, and 2:2), incubated for 10 min at 90 °C, cooling read absorbance at 434 nm. Each point represents the mean of three experiments ± SE.

The proposed method for RS detection was optimized, considering some crucial factors, including gelatin-silver nitrate ratio, time, PH, temperature, and color formation. Maltose was selected as a representative RS for the method optimization. Figure 1 shows the effect of the gelatin-silver ratio on the developed optical density (OD) value that was measured at the SPR of the in situ formed AgNPs. As shown in the figure, the ratio 1:3 w/w of gelatin-silver nitrate ratio was most effective in color formation owing to the in situ AgNPs. The inset color image reveals the intensity of the SPR color of AgNPs.

**Figure 2:**
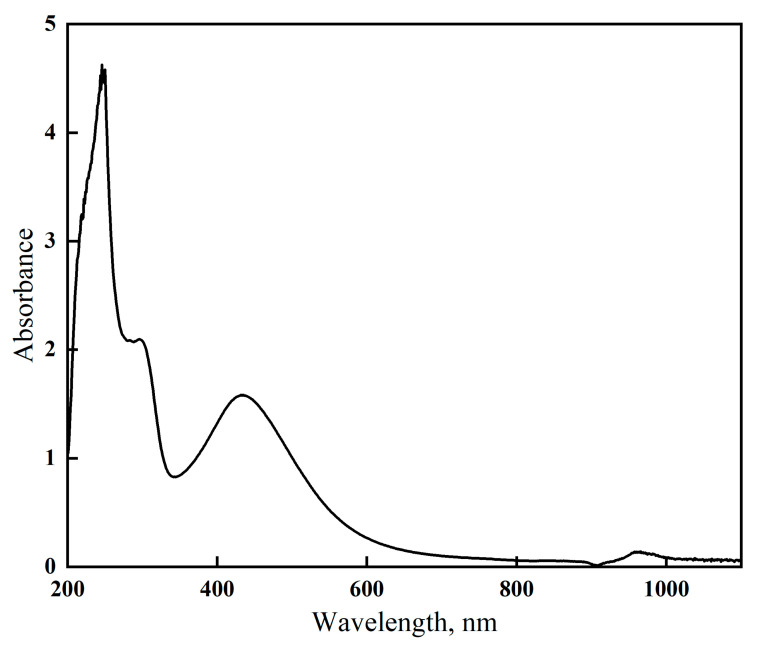
Wavelength of AgNPs produced after gelatin-silver reagent (1:3) react with maltose. Conditions: A 1 mL of the reaction mixture contained 250 µL maltose (0.1 mM), 250 µL Tris–HNO_3_ buffer PH (0.2 M), 500 µL gelatin-silver reagent (1:3), incubated for 10 min at 90 °C.

The UV-visible spectrum of the in situ-formed AgNPs is shown in Figure 2. The SPR peak is displayed at 434 nm to confirm the formation of AgNPs due to the occurrence of a redox reaction between silver nitrate and maltose-reducing sugar. The successful appearance of stable color due to gelatin-capped AgNPs confirms their formation in agreement with other similar green syntheses of AgNPs [33].

**Figure 3:**
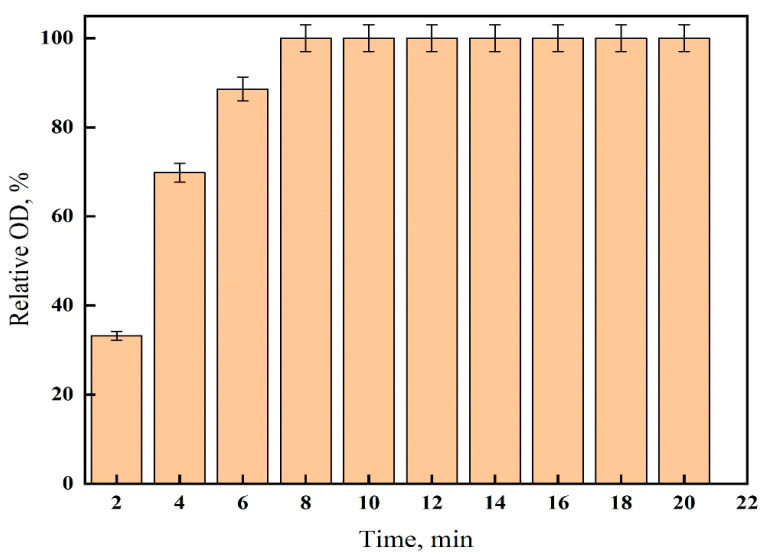
Effect of time on the color evolution of gelatin-silver reagent. Conditions: A 1 mL of the reaction mixture contained 250 µL maltose (0.1 mM), 250 µL of 0.2 M Tris–HNO_3_ buffer PH 8.5, 500 µL gelatin-silver reagent (1:3), incubated for different times at 90 °C in a boiling water bath, cooling, recorded the absorbance at 434 nm. Each point represents the mean of three experiments ± SE.

The effect of time on the evolution of color was monitored spectrophotometrically at the SPR peak. As shown in Figure 3, the development of AgNPs color increases by time up to 8–10 min above, after which there was no further increase, indicating fast response for RS detection in about 10 min. In addition, the OD values remain the same over the studied time, indicating the stability of the in situ-formed AgNPs. Therefore, compared with the conventional DNS method [17], the short-time response and the stability of AgNPs suggest the suitability of the gelatin-silver method for detecting RS.

**Figure 4:**
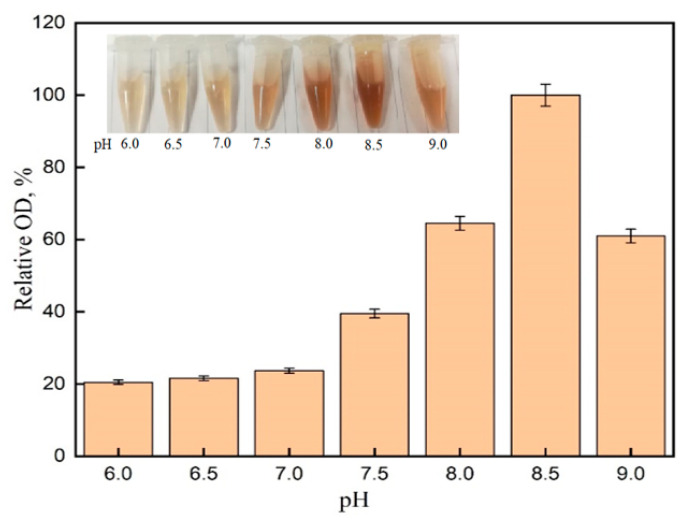
Effect of PH on the color evolution of the gelatin-silver reagent. Conditions: A 1 mL of the reaction mixture contained 250 µL maltose (0.1 mM), 250 µL Tris–HNO_3_ buffer with deferent PH (0.2 M), 500 µL gelatin-silver reagent (1:3), incubated for 10 min at 90 °C, cooling read absorbance at 434 nm. Each point represents the mean of three experiments ± SE.

**Scheme 1:**
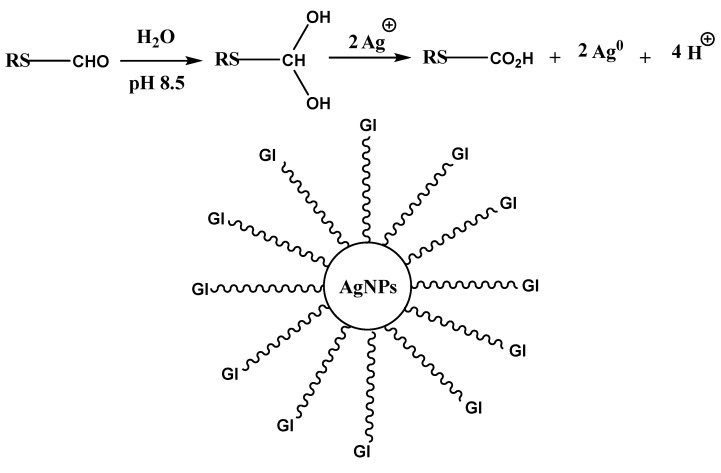
Possible mechanism for the formation of gelatin (Gl) stabilized AgNPs.

The PH of the reaction mixture was varied to obtain the optimum PH. As shown in Figure 4, the formation of AgNPs needs a slightly alkaline medium to form the intensive color of AgNPs. The inset color image reveals the color-PH dependent on the SPR color of AgNPs. The results agree with our previous studies and those found in literature [28,29,37]. Subsequently, PH 8.5 was selected as the optimum value of the gelatin-silver reagent. This alkaline PH is favorable for reducing silver nitrate with RS by enhancing the addition of water molecules on the carbonyl groups, as shown in Scheme 1.

**Figure 5:**
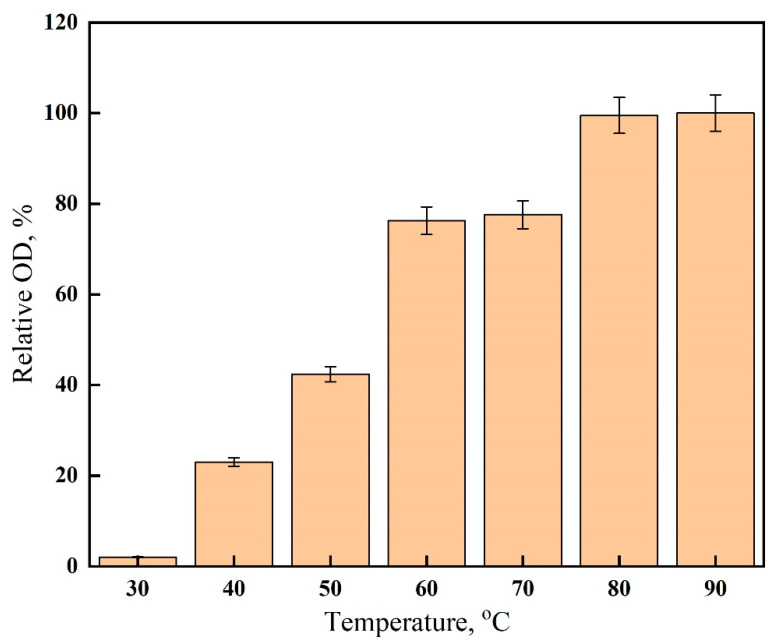
Effect of temperature on the color evolution of gelatin-silver reagent. Conditions: A 1 mL of the reaction mixture contained 250 µL maltose (0.1 mM), 250 µL of 0.2 M Tris–HNO_3_ buffer PH 8.5, 500 µL gelatin-silver reagent (1:3), incubated at different temperature, cooling, recorded the absorbance at 434 nm. Each point represents the mean of three experiments ± SE.

The impact of temperature on AgNPs’ color is depicted in Figure 5. Therefore, the detection of RS works well at a temperature close to 90 °C. As a result, 90 °C was used to conduct the gelatin-silver reagent’s redox reaction.

### 3.2. Possible Mechanism for Gelatin-Capped AgNPs

The presence of the aldehyde-containing compound in an aqueous alkaline medium may lead to the addition of water molecules on the carbonyl group and, subsequently, the formation of an oxysilver complex that ultimately converted to silver nanoparticles and carboxylic-containing compound via a redox reaction. The presence of gelatin help stabilizes the nanoparticles, as shown in Scheme 1.

### 3.3. Maltose Selectivity and Starch Hydrolysis

**Figure 6:**
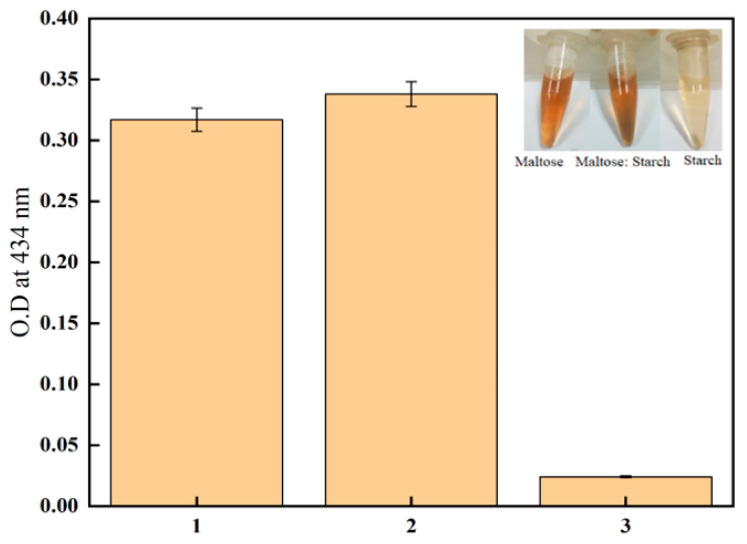
Selectivity for gelatin-silver reagent (1) maltose; (2) maltose: starch; (3) starch. Conditions: A 1 mL of the reaction mixture in sample 1 contained 185 µL of maltose (0.1 mM),190 µL distilled water, 125 µL of Tris-HNO_3_ (0.2 M, PH 8.5); sample 2 contained 185 µL of maltose (0.1 mM), 185 µL of starch (1%), 125 µL of Tris-HNO_3_ (0.2 M, PH 8.5); sample 3 contained 185 µL of starch (1%), 190 µL distilled water, 125 µL Tris-HNO_3_ (0.2 M, PH 8.5); all samples incubated for 10 min at 90 °C, cooling read absorbance at 434 nm. Each point represents the mean of three experiments ± SE.

Three samples were tested to validate whether the gelatin-silver method is selective toward RS. The first sample was maltose, the second was a mixture of maltose and starch, and the third was starch only. To these samples, gelatin-silver reagent, as described in the experimental section, was mixed, and the evolution of the color was tracked. Figure 6 shows the OD values of the samples, and the inset shows an image of their colors. It is indicated that the reagent is selective toward maltose-reducing sugar but not starch.

**Figure 7:**
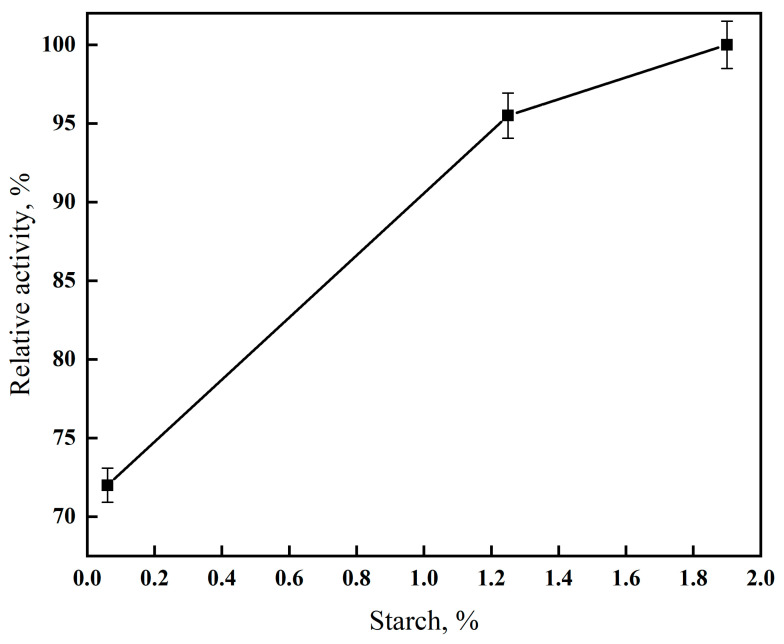
Reduced sugar produced after starch hydrolysis by α-amylase. Conditions: A 1 mL of reaction mixture contained different concentrations of starch (0.06, 1.25, and 1.9%), Tris–HNO_3_ buffers, PH 7.0, incubates at 37 °C, 1 mL of gelatin-silver reagent (1:3), heated for 10 min at 90 °C, recorded absorbance at 434 nm. Each point represents the mean of three experiments ± SE.

Furthermore, starch hydrolysis was made using α-amylase, and the RS obtained was detected by gelatin-silver reagent, as shown in Figure 7. It is shown that the higher the content of starch, the higher the activity of the α-amylase in increasing the production of RS, as evidenced spectrophotometrically by the gelatin-silver reagent.

### 3.4. Limit of Detection of Maltose

**Figure 8:**
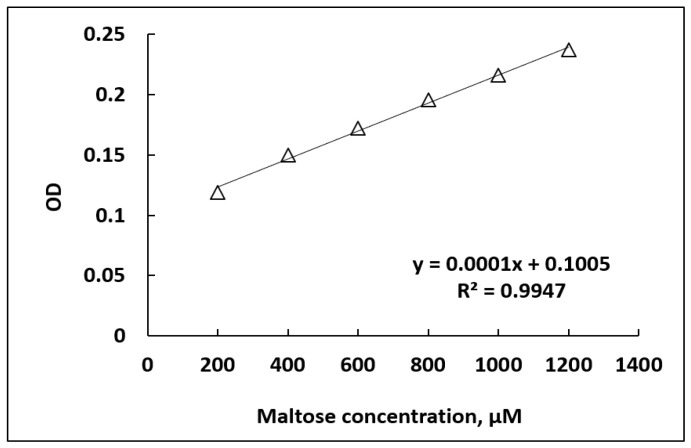
Calibration curve of maltose concentration versus OD. Conditions: See the conditions under Figure 5 using different concentrations of maltose.

The following equation calculated the limit of detection (LOD); LOD = 3.3 σ/S (40 and elsewhere), where σ is the standard deviation of 10 repeated readings of the measured optical density at 434 nm for a selected sample and S is the slope of the calibration curve (Figure 8) of maltose concentration versus OD values. The LOD obtained is LOD = 46.67 µM. Table 1 shows a comparative LOD limit of reported reagents with the present work. Gelatin-silver reagent reveals higher sensitivity than the conventional time-consuming DNS method. As we know, DNS methods are multistep and complicated processes that take more than 1 h. [17].

### 3.5. Real Samples Analysis

Real sample analysis was made for selected commercial samples, namely, honey, watermelon, and fresh apple juice. The total reducing sugars were determined by the conventional DNS method and the developed gelatin-silver method. Table 2 shows that the gelatin-silver method produced similar analysis data made by the DNS method to suggest its viability for food industries.

## 4. Conclusions

This work is devoted to developing a method for measuring reducing sugars using nanoparticles based on silver nitrate and gelatin. The developed approach was improved by considering many essential aspects, including the amount of reagent, reaction interval, PH, temperature, and the gelatin-silver reagent’s selectivity for starch and maltose. For optimization studies, maltose was selected as the representative reducing sugar. The gelatin-silver reagent showed a fast response (less than 10 min) with a detection limit for maltose at 46.67 µM more sensitive than DNS conventional method. Gelatin-silver nitrate in a ratio of 1:3 *w*/*w* produced the best results for color formation. The development of AgNPs color increases within 8–10 min at PH 8.5 as the selected optimum value and at the optimum temperature of 90 °C for the evolution of the gelatin-silver reagent’s redox reaction. In addition, the selectivity of maltose was checked in the presence of starch and after its hydrolysis with α-amylase. Compared with the conventionally used DNS colorimetric method, the proposed method could be applied to commercial fresh apple juice, watermelon, and honey to prove its viability for detecting reducing sugar in food products. The present work explored a viable method for determining the reducing sugar in food industries. Furthermore, the color-based AgNPs would inspire future success in exploiting other colorful nanomaterials to detect reducing sugars. 

## Figures and Tables

**Table 1:** Comparative LOD of reducing sugar with other reported work.

Reference	Method	Analyte	LOD
[17,40]	DNS	Glucose	80 µM
[37]	Tollens’ Reagent	Glucose	40 nM
[41]	DNS	Honey	83.3 µM
[40]	Alkaline Cu(II)-Nc	Glucose	0.6 µM
[42]	Electrochemical Biosensor	Maltose	100 µM
[43]	Benedict’s reagent	Maltose	96.49 µM
[17]	DNS	Maltose	80 µM
This work	Gelatin-silver	Maltose	46.67 µM

**Table 2:** Total reducing sugar content (as mg maltose Eq. g^−1^) of studied samples. Each point represents the mean of three experiments ± SE.

mg Maltose Eq. g^−1^		
Sample	Gelatin-Silver Reagent	DNS
Fresh apple juice	287 ± 1.05	284 ± 0.84
Watermelon	165 ± 0.95	169 ± 0.76
Honey	751 ± 1.12	723 ± 0.92

## Data Availability

All data are presented in the article.
